# 1-Benzyl-3-cetyl-2-methylimidazolium Iodide (NH125) Is a Broad-Spectrum Inhibitor of Virus Entry with Lysosomotropic Features

**DOI:** 10.3390/v10060306

**Published:** 2018-06-05

**Authors:** Sarah Moeschler, Samira Locher, Gert Zimmer

**Affiliations:** 1 Institut für Virologie und Immunologie (IVI), Abteilung Virologie, CH-3147 Mittelhäusern, Switzerland; sarah@moeschler.name (S.M.); samira.locher@ivi.admin.ch (S.L.); 2 Graduate School for Cellular and Biomedical Sciences, University of Bern, CH-3012 Bern, Switzerland

**Keywords:** eukaryotic elongation factor 2 kinase, kinase inhibitor, membrane fusion, alkylated imidazolium, ionic liquid, virus entry, enveloped virus, lysosomotropic agent

## Abstract

Cellular kinases are crucial for the transcription/replication of many negative-strand RNA viruses and might serve as targets for antiviral therapy. In this study, a library comprising 80 kinase inhibitors was screened for antiviral activity against vesicular stomatitis virus (VSV), a prototype member of the family *Rhabdoviridae*. 1-Benzyl-3-cetyl-2-methylimidazolium iodide (NH125), an inhibitor of eukaryotic elongation factor 2 (eEF2) kinase, significantly inhibited entry of single-cycle VSV encoding a luciferase reporter. Treatment of virus particles had only minimal effect on virus entry, indicating that the compound primarily acts on the host cell rather than on the virus. Accordingly, resistant mutant viruses were not detected when the virus was passaged in the presence of the drug. Unexpectedly, NH125 led to enhanced, rather than reduced, phosphorylation of eEF2, however, it did not significantly affect cellular protein synthesis. In contrast, NH125 revealed lysosomotropic features and showed structural similarity with *N*-dodecylimidazole, a known lysosomotropic agent. Related alkylated imidazolium compounds also exhibited antiviral activity, which was critically dependent on the length of the alkyl group. Apart from VSV, NH125 inhibited infection by VSV pseudotypes containing the envelope glycoproteins of viruses that are known to enter cells in a pH-dependent manner, i.e. avian influenza virus (H5N1), Ebola virus, and Lassa virus. In conclusion, we identified an alkylated imidazolium compound which inhibited entry of several viruses not because of the previously postulated inhibition of eEF2 kinase but most likely because of its lysosomotropic properties.

## 1. Introduction

In recent decades an increasing number of chemotherapies have become available for the treatment of viral infections, e.g., drugs with antiviral activity against herpesviruses, HIV-1, influenza A virus, or hepatitis C virus [1]. The majority of antiviral therapies target a specific viral protein, e.g., a component of the viral replication machinery. Drugs belonging to this category are less likely to cause side effects compared to drugs targeting a cellular component, but they may select more easily for drug-resistant mutant viruses. This is in particular true for RNA viruses which possess error-prone RNA polymerases and produce a plethora of quasi species [2]. Furthermore, the currently available antiviral drugs are mostly directed to distinct viral pathogens or small groups of viruses and do not exhibit broad-spectrum antiviral activity [3]. Thus, drugs for the treatment of newly emerging viruses are not readily available.

Viruses essentially rely on cellular factors for replication and some of these factors may represent attractive targets for antiviral therapy. Although there is always the risk of side effects if drugs are targeting host factors, they are less likely to drive the emergence of virus escape mutants. Furthermore, as some viruses may rely on the same cellular pathways for replication, drugs targeting key components of these pathways may result in the discovery of broadly active antiviral compounds.

Cellular protein kinases regulate multiple cellular processes and, therefore, it is not surprising that these enzymes play important roles in the replication of many, if not all, viruses [4,5,6,7,8,9,10]. Accordingly, a recently identified multi-kinase inhibitor demonstrated antiviral activity not only against influenza A virus, but also against vesicular stomatitis virus (VSV) and Newcastle disease virus (NDV) [11]. VSV and NDV belong to the order *Mononegavirales*, which comprises non-segmented negative-strand RNA viruses with a similar genome organization and a similar mode of replication [12,13]. All members of the *Mononegavirales* possess a phosphoprotein (P), which serves as a co-factor for the RNA-dependent RNA polymerase [14]. The extensive phosphorylation of the viral P proteins by host kinases is known to affect transcription/replication of several members of the *Mononegavirales*, including VSV [15,16,17,18], NDV [19], bovine respiratory syncytial virus (BRSV) [20], and Rinderpest virus [21,22,23]. However, there are also exceptions from this rule. For example, phosphorylation of the Sendai virus P protein is dispensable for viral replication [24], and in the case of mumps virus, phosphorylation of the nucleoprotein (N) seemed to be more relevant [25].

In the present study, a collection of 80 kinase inhibitors was screened for antiviral activity against VSV, a prototype virus of the family *Rhabdoviridae* within the *Mononegavirales*. The small compound 1-benzyl-3-cetyl-2-methylimidazolium iodide (NH125) was selected as it showed potent inhibitory activity against VSV. Further analysis of this small compound revealed an unexpected mechanism of inhibition of VSV and other viruses.

## 2. Materials and Methods

### 2.1. Cells

Vero (C1008) and HeLa cells were purchased from the American Type Culture Collection (Manassas, VA, USA) and maintained in Glasgow’s minimal essential medium (GMEM; Life Technologies, Zug, Switzerland) supplemented with 5% fetal bovine serum (FBS, Biowest, Nuaillé, France). Madin-Darby canine kidney (MDCK) cells (type II) (kindly provided by Georg Herrler, TiHo Hannover, Germany) were cultured with Earle’s minimal essential medium (EMEM; Life Technologies) and 5% FBS. Baby hamster kidney (BHK)-21 cells were obtained from the German Cell Culture Collection (DSZM; Braunschweig, Germany) and grown in GMEM with 5% FBS. BHK-G43, a transgenic BHK-21 cell clone expressing the vesicular stomatitis virus (VSV) G glycoprotein in a regulated manner, was maintained as described previously [26]. BSR-T7/5 cells constitutively expressing T7 RNA polymerase [27] were kindly provided by Karl-Klaus Conzelmann, LMU München, Germany, and cultured in GMEM with 5% FBS.

### 2.2. Chemicals

A collection of 80 kinase inhibitors (Tocriscreen kinase inhibitor Toolbox) was purchased from Tocris Bio-Techne AG (Zug, Switzerland). All kinase inhibitors were provided in DMSO at a concentration of 10 mM and stored in aliquots at −20 °C. The compounds 1-dodecyl-3-methylimidazolium iodide, 1-decyl-3-methylimidazolium chloride, 1,3-didecyl-2-methylimidazolium chloride, 1-hexyl-3-methylimidazolium iodide, 1-benzyl-3-methylimidazolium chloride, cycloheximide, rapamycin, chloroquine, brefeldin A, bafilomycin A1, and mifepristone were all purchased from Sigma-Aldrich (Buchs, Switzerland). 

### 2.3. Viruses

The recombinant viral vector VSV*∆G(FLuc) has been described previously [28]. This virus lacks the glycoprotein (G) gene and encodes for the reporter proteins firefly luciferase and green fluorescent protein (GFP). VSV*∆G(sNLuc), a related virus encoding secreted NanoLuc luciferase (sNLuc), has been recently described [29]. Both VSV*∆G(FLuc) and VSV*∆G(sNLuc) were propagated on BHK-G43 helper cells and titrated on BHK-21 cells [26]. A propagation-competent VSV expressing GFP from an extra transcription unit (VSV*) was propagated and titrated on BHK-21 cells [30]. Recombinant VSV*∆G(H5,N1,sNLuc) expressing the sNLuc reporter, along with the envelope glycoproteins HA and NA of the highly pathogenic influenza virus A/chicken/Yamaguchi/7/04 (H5N1), was propagated and titrated on MDCK cells as previously reported [31]. The chimeric virus VSV∆G(EBOV-GP,sNLuc) was generated by replacing the VSV G gene in the VSV* genome with the Ebola virus (variant Mayinga) glycoprotein and the GFP gene with the sNLuc gene according to published procedures [32]. Chimeric VSV∆G(EBOV-GP,sNLuc) expressing sNLuc and the Lassa virus glycoprotein was produced accordingly. The chimeric viruses were propagated on Vero cells and titrated by immunostaining of infected cells as described [31]. A recombinant bovine respiratory syncytial virus (BRSV, strain ATue51908) expressing GFP (BRSV*) was generated by replacing the open reading frame (ORF) of the BRSV glycoprotein (G) gene with the GFP ORF. The virus was produced by transfection of BSR-T7/5 cells with genomic cDNA and expression plasmids encoding for the N. P, M2, and L proteins (kindly provided by Karl-Klaus Conzelmann, Max-von-Pettenkofer Institut, München, Germany) using a published procedure [27,33]. Recombinant BRSV* was propagated and titrated on Vero cells taking advantage of the GFP reporter protein. BTV-8 has been propagated on Vero cells and titrated as described previously [34]. Recombinant Sendai virus (Fushimi strain) expressing the DsRed fluorescent protein was propagated on Vero cells and titrated as reported previously [35].

### 2.4. Testing of Kinase Inhibitors for Antiviral Activity

Vero cells were seeded in 96-well microtiter plates (20,000 cells/well) and cultured for 24 h. All compounds of the TocriScreen Kinase Inhibitor Toolbox were diluted 1:1000 in GMEM medium containing 5% FBS to give a final concentration of 10 µM and incubated with the cells for 2 h at 37 °C and 5% CO_2_. The cells were washed and inoculated for 90 min at 37 °C with VSV*∆G(FLuc) using an m.o.i. of 0.5 ffu/cell. The cells were washed again and incubated for 6 h with GMEM medium containing the respective inhibitor. Finally, 30 µL of cell lysis buffer (Promega, Madison, WI, USA) was added to each well and 6 µL of lysate transferred to a white 96-well microtiter plate. Firefly luciferase substrate (Promega) was automatically injected (30 µL/well) into each well and luminescence recorded for 1 s with the Centro LB 960 luminometer (Berthold Technologies, Bad Wildbad, Germany). All kinase inhibitors were subject to at least two rounds of analysis. Those compounds that led to suppression of luciferase activity by at least 70% were selected for a third round of analysis. In some experiments, viruses expressing the sNLuc reporter protein were used. Analysis of sNLuc activity in the cell culture supernatant was performed as described previously [29]. 

### 2.5. Foci-Forming Unit Reduction Assay

BHK-21 cells grown in 96-well cell culture plates were treated for 60 min at 37 °C with 50 µL/well of GMEM medium containing 2.5% FBS and serially diluted NH125 (0.25–10 µM). Medium containing DMSO (0.1%, *v*/*v*) served as the control. Subsequently, 50 µL of GMEM containing 100 ffu infectious virus particles were added to each well and incubated at 37 °C for 60 min. The cells were washed two times with 200 µL/well of GMEM and then again received 50 µL of GMEM containing the respective inhibitor. Following incubation for 60 min 50 µL of GMEM containing 2% FBS and 1.8% (*w*/*v*) methylcellulose were added to each well and incubated for 20 h at 37 °C. In the case of VSV* and BRSV* infectious cell foci could be detected by fluorescence microscopy. In the case of BTV-8 and SeV, the cells were fixed with 3% paraformaldehyde and infected cell foci detected by immunostaining as previously described [34,35].

### 2.6. Western Blot Analysis

Confluent HeLa cells grown in 6-well plates were treated for 6 h with either DMSO, NH125, or rapamycin. The cells were washed twice with phosphate-buffered saline (PBS) and incubated at 37 °C with 250 µL/well of trypsin/EDTA solution (Life Technologies Europe, Zug, Switzerland). Treatment with trypsin was stopped by addition of MEM/5% FBS (250 µL/well). The cells were suspended by repeated pipetting, transferred to 1.5-mL tubes, and spun at 250× *g* for 5 min. The pelleted cells were washed once with PBS and then lysed with 100 µL of cell lysis buffer (New England Biolabs, Herts, UK) containing a cocktail of proteinase and phosphatase inhibitors (Sigma-Aldrich). An equal volume of two-fold concentrated SDS sample buffer containing 10% (*v*/*v*) 2-mercaptoethanol (Sigma-Aldrich) was added and the samples were heated for 5 min at 90 °C. The proteins were separated by 10% polyacrylamide gel electrophoresis, and transferred to Porablot 0.45 µm pore size nitrocellulose membranes (Macherey-Nagel, Oensingen, Switzerland) by semidry blotting (0.8 mA/cm^2^, 60 min). The membrane was incubated for 1 h with blocking reagent (LI-COR Biosciences, Bad Homburg, Germany), washed three times with PBS containing 0.1% Tween 20 (AppliChem, Darmstadt, Germany), and incubated overnight at 4 °C with Tris-buffered saline (TBS, pH 7.2) containing 5% bovine serum albumin (BSA), 0.1% Tween 20 and antibodies directed to either eEF2 or phosphorylated eEF2 (1:1000 each). The nitrocellulose membrane was washed three times with PBS/0.1% Tween 20 and incubated for 1 h at room temperature with IRDye^®^ 800CW goat anti-rabbit IgG diluted 1:10,000 in TBS/5% BSA/0.1% Tween 20. The nitrocellulose membrane was washed three times with PBS/0.1% Tween 20 and once with detergent-free PBS. The proteins were detected using the Odyssey Infrared Imaging System (LI-COR Biosciences, Bad Homburg, Germany).

### 2.7. Syncytia Formation

BHK-G43 cells were seeded into 24-well plates containing 12-mm glass coverslips and cultured for 24 h with GMEM containing 5% FBS. The nearly confluent cells received fresh cell culture medium containing mifepristone (10^−9^ M) and either NH125 (1–10 µM), bafilomycin A1 (0.08 µM), or DMSO (0.1%, *v*/*v*), and were maintained for 24 h at 37 °C with 5% CO_2_. The cells were washed once with PBS (4 °C) and fixed for 30 min at room temperature with 3% paraformaldehyde (AppliChem, Darmstadt, Germany). The cells were washed twice with PBS containing 0.1 M glycine (AppliChem) and once with PBS. Subsequently, the cells were incubated with a mouse monoclonal antibody (hybridoma clone I1, American Type Culture Collection, Manassas, VA, USA) directed to the VSV-G protein (1:50), washed three times with PBS, and then incubated for 1 h at room temperature with a goat anti-mouse IgG AlexaFluor-488 conjugate (1:500, Life Technologies Europe). The cells were rinsed twice with PBS and once with distilled water and the nuclei stained for 5 min with 0.1 µg/mL of diamidino-2-phenylindole (DAPI; Sigma) dissolved in ethanol. Finally, the cells were washed with distilled water and embedded in Mowiol 4–88 (Sigma) mounting medium. 

### 2.8. Quantitative Fusion Assay

The quantitative fusion assay took advantage of the commercially available NanoBiT^®^ protein interaction system (Promega, Madison, WI, USA). BHK-G43 cells grown in six-well plates were separately transfected with expression plasmids encoding for either the catalytic subunit α of protein kinase A genetically linked to the small fragment of NanoLuc luciferase (SmBiT-PRKACA) or the cAMP-dependent protein kinase type II α regulatory subunit fused to the large fragment of NanoLuc luciferase (LgBiT-PRKAR2A). Twenty-four hours post transfection, the two cell populations were suspended with trypsin, mixed at equal parts giving a suspension of 10^6^ cells/mL, and then seeded in 96-well microtiter plates (100 µL/well). Four hours after seeding, the cell culture medium was replaced by GMEM containing 5% FBS, 10^−9^ M of mifepristone (to induce VSV G protein expression), and the inhibitor to be tested. After incubating the cells at 37 °C for 24 h, the cell culture supernatant was aspirated, the cells incubated for 15 min at 37 °C with 25 µL of trypsin-EDTA solution (Life Technologies Europe, Zug, Switzerland) and suspended with 75 µL of Opti-MEM medium (Life Technologies Europe). To each well 25 µL of Nano-Glo^®^ live cell reagent (Promega) was added and luminescence recorded for 10 s. Transfected BHK-G43 cells that were not induced with mifepristone served as a negative control for the VSV G protein-mediated membrane fusion leading to functionally complementation of the NanoLuc luciferase fragments LgBiT and SmBiT. 

### 2.9. Cytotoxicity Assay

BHK-21 cells were seeded in 96-well white cell culture plates (20,000 cells/well) and cultured with GMEM/5% FBS medium for 24 h at 37 °C. The medium was replaced with fresh medium (100 µL/well) containing either DMSO (0.1%, *v*/*v*) or NH125 (10 µM, 5 µM or 2.5 µM). At the indicated time points, 50 µL of CytoTox-Glo^TM^ cytotoxicity assay reagent (Promega) were added to each well and incubated for 15 min at room temperature before luminescence was recorded for 0.5 s. Thereafter 50 µL of digitonin lysis reagent (Promega) were added to each well, and incubated for 15 min with shaking before luminescence was recorded. The percentage of viable cells was calculated for quadruplicate wells for each time point.

### 2.10. In Vitro Translation Assay

The plasmid pCDNA3.1-FLuc encoding firefly luciferase was linearized with the restriction endonuclease PvuII downstream of the poly-A sequence. The linearized DNA (10 µg) served as template for the in vitro transcription and capping of the messenger RNA with the mMESSAGE mMACHINE Ultra T7 kit (ThermoFisher) and the m7G(5′)ppp(5′)G cap analog, respectively. The reaction mixture was treated with DNase I to remove the template DNA and subsequently purified using Illustra MicroSpin S-400 HR GE healthcare columns (Sigma-Aldrich, Buchs, Switzerland). The transcribed RNA (2 µg) was translated in vitro into enzymatically-active firefly luciferase using the rabbit reticulocyte lysate system (Promega, Madison, WI, USA) according to the manufacturer’s instructions. The reaction was performed in the presence of either 10 µM of NH125 or DMSO. The firefly luciferase enzyme test was performed as described above.

### 2.11. Statistical analysis

Mean values and standard deviations (SD) were calculated. Data were analyzed by Student’s *t*-test and *P* < 0.05 was considered as significant (indicated by asterisks). 

## 3. Results

### 3.1. Identification of a Small Compound Showing Antiviral Activity Against VSV

**Figure 1:**
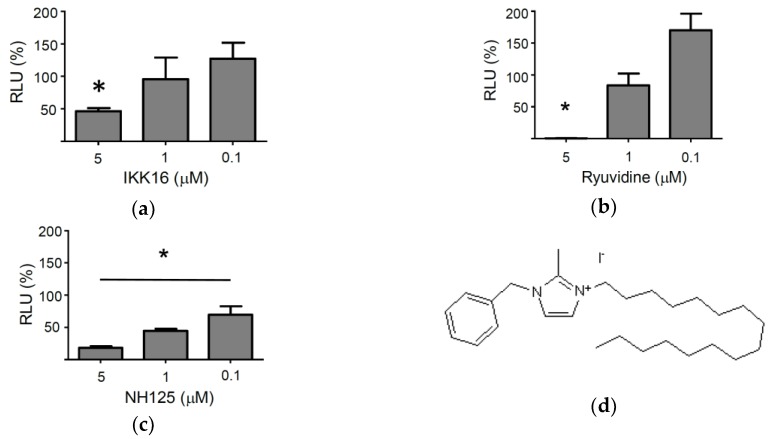
Effect of selected kinase inhibitors on VSV*ΔG(FLuc)-driven reporter gene expression. The three compounds IKK16 (**a**), ryuvidine (**b**) and NH125 (**c**), which showed >95% suppression of VSV-driven firefly luciferase reporter expression when used at 10 µM (supplementary Table S1), were tested for antiviral activity using lower concentrations (5 µM, 1 µM, and 0.1 µM). Vero cells were treated with the compounds at the indicated concentration for 2 h, inoculated for 1 h with VSV*∆G(FLuc) using an m.o.i. of 0.5 and then further incubated with the indicated compounds for six more hours. Luminescence emission was determined in cell lysates for 1 s using luciferin as substrate and expressed as relative luminescence units (RLU). Percentage RLU was calculated using the luminescence emission of DMSO-treated cells as reference. Mean values and standard deviations of three infection experiments are shown. Asterisks indicate significant inhibition of infection (compared to DMSO-treated control cells). (**d**) Chemical structure of NH125 [36].

A collection of 80 kinase inhibitors (TocriScreen Kinase Inhibitor Toolbox) was screened for antiviral activity against VSV*∆G(FLuc), a propagation-incompetent, envelope glycoprotein (G) gene-deleted virus which encodes for the firefly luciferase reporter protein (FLuc) [26]. VSV*∆G(FLuc) was produced on genetically-engineered helper cells providing the VSV-G protein in trans [26]. The *trans*-complemented virus replicon particles (VRP) have previously been shown to infect permissive cells and to perform transcription and replication in the cytosol, while being unable to produce progeny virus [28]. The VRP system may allow detection of antiviral compounds that interfere with virus entry or with viral transcription/replication, but will not detect compounds that affect virus assembly or release. The screening of the kinase inhibitors for antiviral activity was performed with Vero cells which are unable to produce type I interferon. Thus, any antiviral effects that may be observed may not be due to the induction and release of type I interferons. The Vero cells were treated with the individual kinase inhibitors (10 µM) for 1 h and then inoculated for 90 min with VSV*∆G(FLuc) using a multiplicity of infection (m.o.i.) of 0.5 ffu/cell. The cells were washed and then maintained for 6 h in the presence of the respective compounds. Finally, the cells were lysed and firefly luciferase activity recorded. Of the 80 kinase inhibitors analyzed three compounds were able to suppress expression of the virus-encoded reporter by more than 95%: NH125, ryuvidine, and IKK16 (supplementary Table S1). However, only NH125, a CAM kinase III inhibitor, also showed inhibitory activity at concentrations below 5 µM (Figure 1).

### 3.2. NH125 Inhibits Viral Entry by Acting on the Host Cell

**Figure 2:**
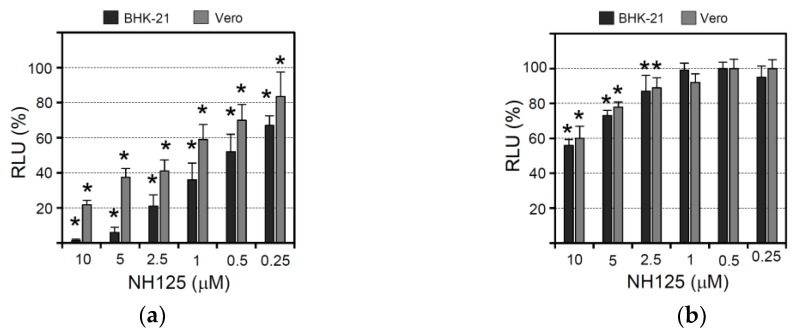

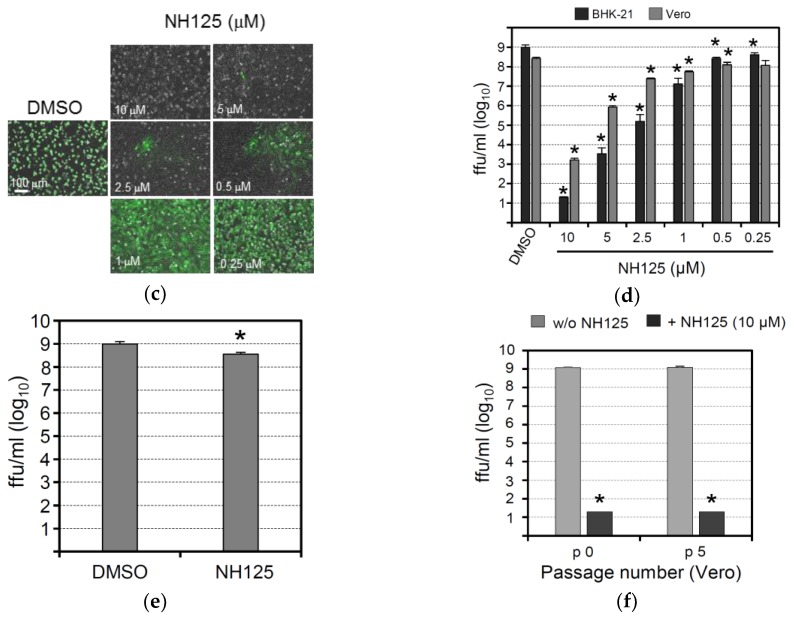
Effect of NH125 on virus-driven luciferase expression and infectious VSV titers. (**a**) Vero and BHK-21 cells were treated for 1 h with NH125 using the indicated concentrations, washed with medium, and subsequently inoculated for 1 h with VSV*∆G(sNLuc) using an m.o.i. of 0.5 ffu/cell. Virus which had not entered the cells during the incubation period was neutralized with a monoclonal antibody directed to the VSV G protein. Secreted NanoLuc luciferase activity was determined in the cell culture supernatant 6 h p.i. The percentage RLUs relative to DMSO-treated VSV*∆G(sNLuc)-infected cells were calculated. Mean values and standard deviations of six parallel experiments are shown. Asterisks indicate significant inhibition of virus entry (compared to DMSO-treated cells). (**b**) Vero and BHK-21 cells were inoculated for 6 h with VSV*∆G(sNLuc) (m.o.i. of 0.5 ffu/cell) and washed to remove sNLuc reporter protein which has been secreted up to this time point. Subsequently, the cells were incubated for 5 h with NH125 at the indicated concentrations. Luciferase activity was determined as above. (**c**) BHK-21 cells treated for 2 h with either DMSO (0.1%, *v*/*v*) or NH125 using the indicated concentrations and subsequently infected for 1 h with VSV* (m.o.i. of 0.001 ffu/mL) and subsequently maintained for 24 h in the presence of NH125 or DMSO. GFP fluorescence indicating infected cells was detected by fluorescence microscopy. (**d**) Infectious VSV* titers released into the cell culture supernatant of infected BHK-21 and Vero cells following treatment with the indicated concentrations of NH125. Mean titers and standard deviations of three infection experiments are shown and expressed as fluorescent focus-forming units per ml (ffu/mL). (**e**) VSV* was treated at 37 °C for 2 h with either DMSO or NH125 (10 µM) prior to ultracentrifugation. Infectious virus titers were subsequently determined on BHK-21 cells. Mean titers and standard deviations of three independent experiments are shown. (**f**) VSV* was passaged on Vero cells in the presence of NH125 (10 µM). The infectivity of passage 5 virus compared to non-passaged VSV was determined on BHK-21 cells that have been treated with NH-125 (10 µM).

In order to determine whether NH125 would show inhibitory activity if the cells were treated before, but not during or after virus infection, Vero and BHK-21 cells were incubated for 1 h with NH125 and then inoculated for 1 h (in the absence of NH125) with VSV*∆G(sNLuc), a propagation-defective G gene-deleted VSV expressing secreted NanoLuc luciferase (sNLuc). Subsequently, the cells were incubated with medium containing a VSV-neutralizing antibody in order to limit the virus entry phase to 1 h. The activity of the sNLuc reporter protein secreted into the cell culture medium was determined at 6 h post infection. It turned out that NH125 suppressed reporter activity in a concentration-dependent manner in both Vero and BHK-21 cells (Figure 2a). The most pronounced effect was found with 10 µM of NH125 which led to a twenty-fold (95%) reduction of sNLuc activity. The IC_50_ values for NH125-mediated inhibition of infection of BHK-21 and Vero cells were about 0.5 µM and 1 to 2.5 µM, respectively, indicating that BHK-21 cell are more sensitive to NH125 action than Vero cells. Our results also suggest that pretreatment of cells with NH125 is already sufficient to inhibit virus infection.

In order to see whether NH125 would also have an impact on a later step of the viral replication cycle, Vero and BHK-21 cells were first infected with VSV*∆G(sNLuc) (m.o.i. of 0.5 ffu/cell) for 6 h and then washed in order to remove all sNLuc produced and secreted up to this time. Subsequently, the cells were incubated for five more hours in the presence of NH125 at different concentrations. In this experimental setting NH125 had only a minor suppressive effect on reporter activity with 10 µM of NH125 leading to about a 40% reduction of luciferase activity (Figure 2b). This finding suggests that NH125 primarily affects an early step in the viral replication cycle. 

To study the effects of NH125 when present during the whole VSV replication cycle, Vero and BHK-21 cells were first treated for 2 h with serially diluted NH125 (starting with 10 µM) and subsequently inoculated (m.o.i. of 0.001 ffu/cell) with a propagation-competent recombinant VSV expressing GFP (VSV*). Virus adsorption was allowed to take place for 60 min before the cells were washed and incubated with NH125 for 21 h allowing the virus to perform about three replication cycles. Detection of GFP-positive cells by fluorescence microscopy then revealed that NH125 inhibited infection in a concentration-dependent manner with 10 µM of NH125 completely abrogating infection (Figure 2c). Titration of infectious VSV* released into the cell culture supernatant showed that infectious virus titers dropped by more than 8 log_10_ when NH125 was used at 10 µM and by 2 log_10_ when using NH125 at 1 µM (Figure 2d). The IC_50_ and IC_90_ values of NH125 on BHK-21 cells were about 0.25 µM and 0.5 µM, respectively. In Vero cells, 10 µM of NH125 reduced virus titers by 5 log_10_ while 1 µM reduced the virus titer five-fold, indicating that treatment of Vero cells with NH125 is less efficient compared to BHK-21 cells.

To figure out whether NH125 would directly affect the infectivity of virus particles, a VSV* suspension containing 10^8^ infectious particles per mL was treated for 2 h at 37 °C with 10 µM of NH125. The virus was pelleted through a sucrose cushion to remove the drug titrated on BHK-21 cells. It turned out that NH125 reduced virus titers three-fold (Figure 2e), suggesting that the drug essentially exerts effects on the host cell rather than on the virus. VSV* remained fully sensitive to the action of NH125 even after five passages on Vero cells in the presence of 10 µM of NH125 (Figure 2f).

### 3.3. Analysis of the Antiviral Activity of NH125-Related Imidazolium Derivatives

**Figure 3:**
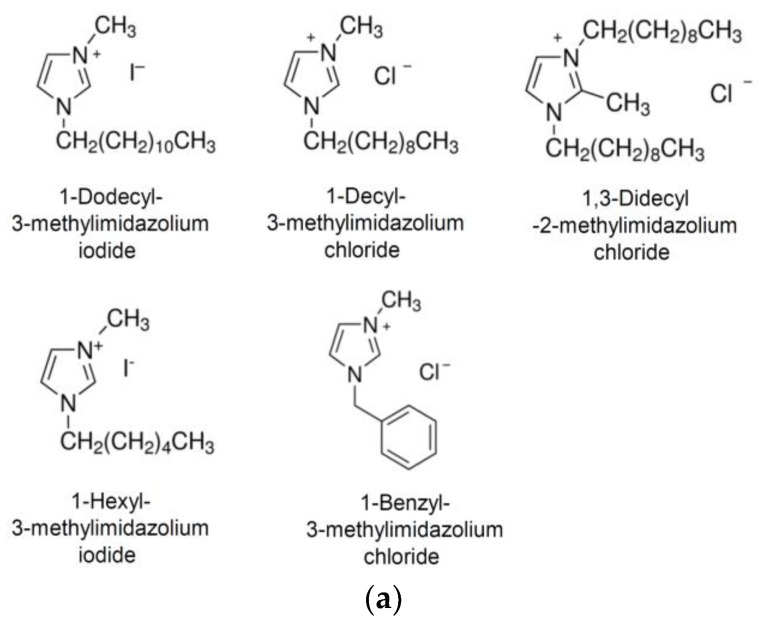

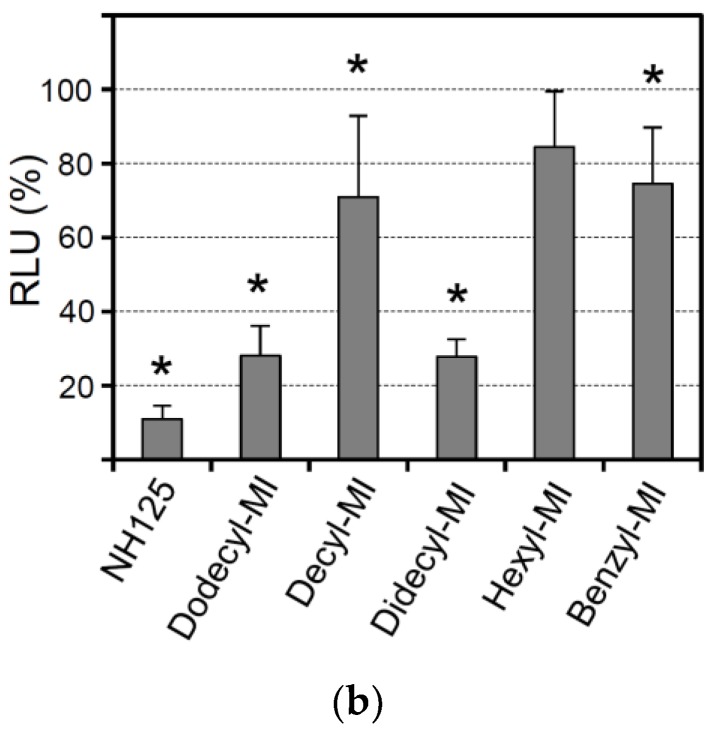
Antiviral activity of NH125-related imidazolium derivatives. (**a**) Chemical structures of 1-dodecyl-3-methylimidazolium iodide, 1-decyl-3-methylimidazolium chloride, 1,3-dicyl-2-methylimidazolium chloride, 1-hexyl-3-methylimidazolium iodide, and 1-benzyl-3-methylimidazolium chloride. (**b**) Vero cells were treated for 1 h with the indicated compounds (10 µM) and subsequently infected with VSV*ΔG(sNLuc) as described in the legend for Figure 2. Secreted NanoLuc luciferase activity was determined in the cell culture supernatant 6 h p.i. Mean values and standard deviations of three experiments are shown. Asterisks indicate significant inhibition of virus infection compared to DMSO-treated cells.

NH125 is an imidazolium derivative which is substituted at positions 1, 2, and 3 with a benzyl, methyl, and cetyl groups, respectively [36]. To see whether the substituents would be important for antiviral activity, related imidazolium compounds with different side groups were investigated (Figure 3a). When the cells were pretreated with 1-dodecyl-3-methylimidazolium iodide (Dodecyl-MI), infection with VSV*∆G(sNLuc) was reduced, although not to the same extent compared to NH125 (Figure 3b). The imidazolium derivative 1-decyl-3-methylimidazolium chloride (Decyl-MI) with a shorter aliphatic side chain (Figure 3b) was less active compared to Dodecyl-MI while 1,3-didecyl-2-methylimidazolium chloride (Didecyl-MI) containing two dodecyl side groups was as active as Dodecyl-MI. Imidazolium derivatives with either a shorter aliphatic side chain (1-hexyl-3-methylimidazolium iodide, Hexyl-MI) or without such a side group (1-benzyl-3-methylimidazolium chloride, Benzyl-MI) (Figure 3b) showed no, or low, antiviral activity. These findings suggested that NH125-related imidazolium derivatives may also exhibit antiviral activity, which critically depends on the length of the aliphatic side chain.

### 3.4. Effects of NH125 on Elongation Factor 2 Phosphorylation and Protein Synthesis

**Figure 4:**
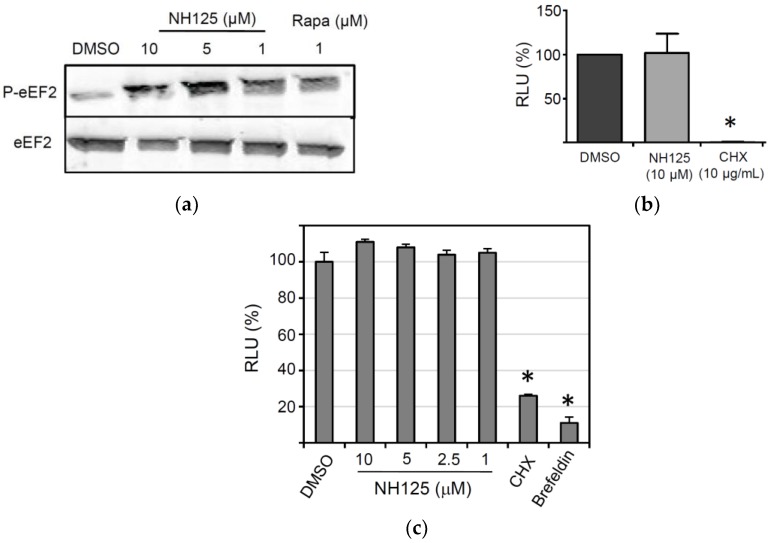
Impact of NH125 on eEF2 phosphorylation and protein synthesis. (**a**) Detection of eEF2 phosphorylation in HeLa cells. The cells were treated for 8 h with NH125, rapamycin or DMSO prior to lysis and Western blot analysis with antibodies directed to eEF2 and phosphorylated eEF2 (P-eEF2). (**b**) Effect of NH125 on in vitro translation of firefly luciferase mRNA. In vitro transcribed luciferase mRNA was incubated for 2 h at room temperature with rabbit reticulocyte lysates in the presence of either NH125 (10 µM), DMSO (0.1%, *v*/*v*) or cycloheximide (CHX; 10 µg/mL). Firefly luciferase activity was determined with luciferin as the substrate and expressed as the percentage RLU (relative to the DMSO control). Mean values and standard deviations of three in vitro translation experiments are shown. (**c**) BSR-T7/5 cells grown in 24-well plates were transfected with the plasmid pTM1-sNLuc (0.5 µg/well) and incubated for 6 h at 37 °C. The cells were washed and incubated for 16 h with medium containing either DMSO or NH125 at the indicated concentrations. The inhibitors cycloheximide (10 µg/mL) and brefeldin A (5 µg/mL) were used as controls. Secreted sNLuc activity was determined in the cell culture supernatant as described above. Mean values and standard deviations of three transfection experiments are shown. Asterisks indicate significantly different reporter activity compared to DMSO-treated control cells.

NH125 has previously been described to act as an inhibitor of the eukaryotic elongation factor 2 (eEF2) kinase, a calmodulin-dependent kinase that inactivates eEF2 through phosphorylation of threonine at position 56 [37]. However, there is also evidence showing that NH125 actually promotes phosphorylation of eEF2 [38,39]. To study the effect of NH125 on eEF2 phosphorylation, HeLa cells were treated with NH125 for 8 h before the cell lysates were analyzed by Western blot. We observed that NH125 increased phosphorylation of eEF2, but had no apparent effect on the overall eEF2 content if used at 5 µM or lower concentrations (Figure 4a). Rapamycin, which has previously been shown to induce phosphorylation of eEF2 [40,41], was used as a positive control (Figure 4a). To address if NH125 has any impact on protein synthesis, an *in vitro* translation assay was performed. Rabbit reticulocyte lysates were pre-incubated with either NH125 (10 µM), cycloheximide (CHX, 10 µg/mL) or DMSO (0.1%, *v*/*v*) for 2 h before addition of *in vitro* transcribed and capped mRNA encoding luciferase. We found that NH125 did not inhibit the translation of luciferase mRNA (Figure 4b), suggesting that the antiviral properties of NH125 did not rely on the inhibition of protein synthesis. Next, we tested whether NH125 would affect plasmid-driven expression of a reporter protein. To this end, we transfected BSR-T7/5 cells, a cell line constitutively expressing the T7 phage RNA polymerase [27], with pTM1-sNLuc, a plasmid encoding the sNLuc gene under control of the T7 promotor and an internal ribosome entry site from the encephalomyocarditis virus. Six hours post transfection, the cells were washed to remove all sNLuc which has been secreted until this time, and subsequently incubated the cells for 18 h with either NH125, cycloheximide or brefeldin A. Analysis of the cell culture supernatant revealed that NH125 did not affect the expression of the reporter protein (Figure 4c), in striking contrast to cycloheximide, a drug affecting protein synthesis, and brefeldin A, a compound disturbing the integrity of the secretory pathway [42]. Together, these findings suggest that NH125 does not interfere with cellular protein synthesis nor does it inhibit protein secretion.

### 3.5. NH125 Inhibits VSV G Protein-Mediated pH-Dependent Membrane Fusion

**Figure 5:**
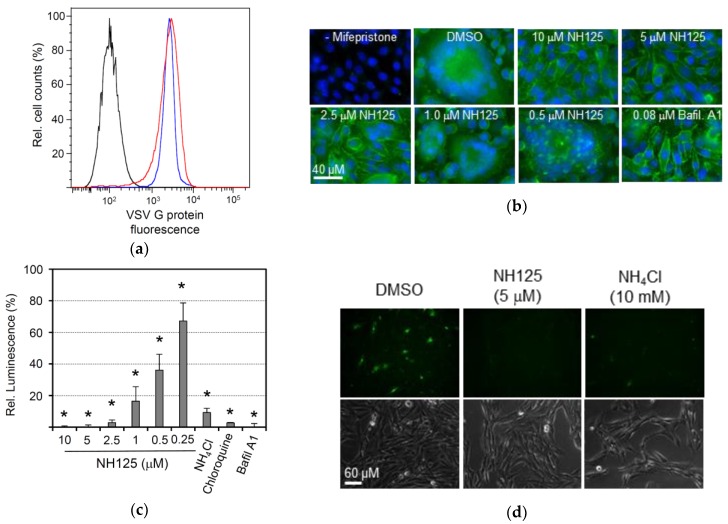
NH125 inhibits VSV G protein-mediated pH-dependent membrane fusion. (**a**) Flow cytometric analysis of VSV G protein cell surface expression in the presence of NH125. BHK-G43 cells were treated with mifepristone to induce VSV G protein expression and maintained for 16 h in the presence of either DMSO (red line) or 10 µM of NH125 (blue line). VSV G protein expression was detected at the cell surface using monoclonal antibody I1 and anti-mouse IgG AlexaFluor-488 conjugate. Non-induced BHK-G43 cells served as a negative control (black line). (**b**) VSV G protein-mediated syncytia formation. BHK-G43 cells were grown on 12-mm glass coverslips. When the cells were grown to confluence, the medium was replaced with fresh medium containing NH125 at the indicated concentrations and mifepristone to induce VSV G protein expression. At 24 h post induction, the cells were fixed with paraformaldehyde and VSV glycoprotein detected by indirect immunofluorescence using the monoclonal antibody I1 and AlexaFluor 488-labelled secondary antibody directed to mouse IgG. Nuclei were stained with DAPI. (**c**) Quantification of VSV G protein-mediated membrane fusion using the NanoBiT^®^ protein interaction system as described in the Methods section. Mean titers and standard deviations of three experiments are shown. Asterisks indicate significantly different inhibition of membrane fusion compared to DMSO-treated cells. (**d**) Analysis of the lysosomotropic features of NH125. BHK-21 cells were first treated for 1 h with either NH_4_Cl, NH125, or DMSO and, subsequently, with pHrodo green dextran which emits fluorescence following uptake into an acidic intracellular compartment.

A transgenic BHK-21 cell clone that expresses the VSV glycoprotein G in a regulated manner has previously been established [26]. In accordance with our findings presented in the previous section, cell surface expression of VSV G protein in this cell line was not affected by NH125 (Figure 5a). However, we observed that VSV G protein-mediated syncytia formation was completely abolished in the presence of 10 µM or 5 µM of NH125, while lower concentrations of NH125 reduced syncytia formation (Figure 5b). Bafilomycin A1, a highly potent inhibitor of vacuolar-type H^+^-ATPase [43], also inhibited syncytia formation, thus confirming the previous notion that the fusion activity of VSV G protein is triggered by the acidic milieu of the Golgi during transport of the glycoprotein to the cell surface via the secretory pathway [44]. To quantify inhibition of VSV G protein-induced membrane fusion we took advantage of a split NanoLuc luciferase reporter assay. To this end, BHK-G43 cells were separately transfected with expression plasmids encoding either the catalytic subunit α of protein kinase A which was genetically linked to the small fragment of NanoLuc luciferase (SmBit-PRKACA) or the cAMP-dependent protein kinase type II α regulatory subunit fused to the large fragment of NanoLuc luciferase (LgBiT-PRKAR2A). One day following transfection, the two cell populations were suspended with the help of trypsin and then seeded together in 96-well microtiter plates. Expression of VSV G protein was induced by the addition of mifepristone. VSV G protein-mediated membrane fusion between the two cell populations allowed the interaction of PRKACA with PRKAR22A and, thereby, led to the complementation of enzymatically-active NanoLuc luciferase. Using this approach, NH125 was found to inhibit membrane fusion in a concentration-dependent manner with an IC_50_ ranging between 0.25 and 0.5 µM (Figure 5c). Compounds that are known to increase the pH value in intracellular compartments, such as NH_4_Cl, choroquine and bafilomycin A1, also inhibited VSV G protein-mediated membrane fusion. In line with this observation, NH_4_Cl and chloroquine inhibited infection of BHK-21 cells with VSV*∆G(sNLuc) in a concentration-dependent manner (Figure S1).

Since NH125 is structurally related to *N*-dodecylimidazole, a compound with lysosomotropic features [45], we wondered whether NH125 would be able to raise the pH in the endocytic pathway. To address this question, BHK-21 cells were first treated for 1 h with either NH125 or NH_4_Cl and subsequently incubated for 15 min with pHrodo green dextran. This molecule is linked to a pH-sensitive fluorescent dye which emits fluorescence only if the compound has entered an acidic compartment, such as the lysosome. Indeed, the cells showed green fluorescence following treatment with DMSO, whereas treatment of the cells with either NH125 or NH_4_Cl prevented emission of fluorescence (Figure 5d). These findings suggest that NH125 has lysosomotropic features similar to NH_4_Cl and chloroquine. 

### 3.6. Analysis of NH125 Cytotoxicity

**Figure 6:**
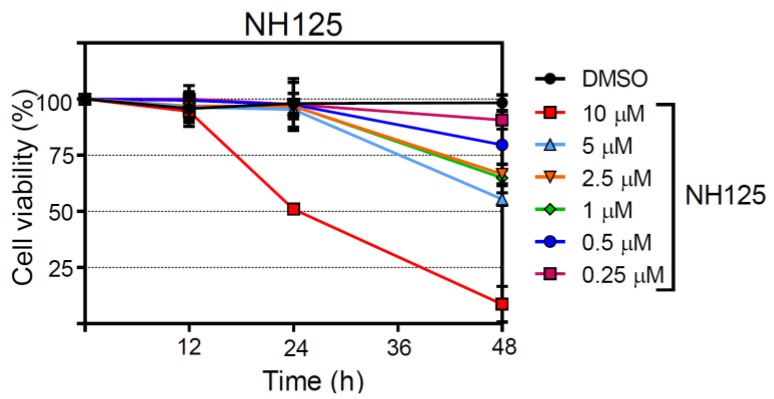
Assessment of the cytotoxic properties of NH125. BHK-21 cells were incubated with either DMSO or NH125 using the indicated concentrations. The proportion of dead cells was determined at the indicated times by determining the activity of dead cell protease released from dying cells and in total cell lysates. Mean values and standard deviations of 4 parallel experiments are shown.

In order to assess the impact of NH125 on cell viability, the release of a protease from dying cells was determined taking advantage of a luminescent protease substrate. NH125 did not reveal cytotoxic effects at any concentration when incubated with BHK-21 for 12 h (Figure 6). However, NH125 killed about 50% of the cells if present for 24 h at a concentration of 10 µM and further reduced cell viability when inoculated with the cells for 48 h. When NH125 was used at concentrations below 10 µM, no cytotoxic effects were observed up to 24 h. However, some cell death was caused by low-concentrated NH125 when the drug was present for 48 h. For comparison, chloroquine also caused cell death in a concentration- and time-dependent manner (Figure S2), indicating that lysosomotropic drugs may interfere with cell viability if used for a prolonged time at too high a concentration. In line with our observations, the cytotoxicity of *N*-dodecylimidazole has also been ascribed to the lysosomotropic features of this NH125-related compound [45,46,47]. 

### 3.7. Broad-Spectrum Antiviral Activity of NH125

**Figure 7:**
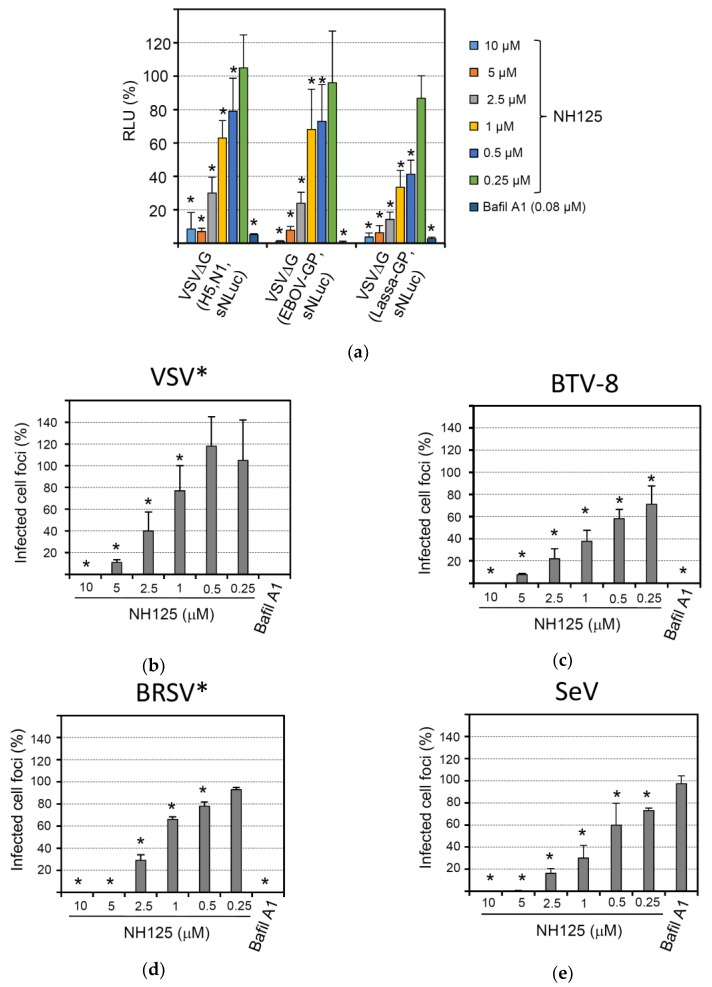
Broad-spectrum antiviral activity of NH125. BHK-21 cells were treated for 1 h with the NH125 using the indicated concentrations or with bafilomycin A1 (0.08 µM). (**a**) The cells were inoculated for 1 h with the indicated chimeric viruses (m.o.i. of 0.05 ffu/mL) in the presence of the inhibitor, washed and maintained in medium for five more hours. Secreted NanoLuc luciferase activity in the cell culture supernatant was determined with a luminescent substrate. The relative luminescence with respect to DMSO-treated control cells is shown. Mean titers and standard deviations of six parallel experiments are shown. (**b**–**e**) NH125-treated BHK-21 cells were inoculated for 1 h in the presence of inhibitor with 100 ffu/well of either VSV* (**b**), BTV-8 (**c**), BRSV* (strain ATue51908) (**d**), or Sendai virus (SeV, strain Fushimi) (**e**). The cells were washed and maintained for 20 h in medium with inhibitor and methylcellulose. The number of infected cell foci was determined and expressed percentage infected cell foci relative to DMSO-treated cells. Mean titers and standard deviations of 6 parallel experiments are shown. Asterisks indicate significantly reduced number of cell foci compared to the DMSO control.

Several enveloped viruses require the acidic milieu of the endocytic compartment for successful infection of the host cell. This is because either the viral fusion activity is triggered by low pH or the viral glycoprotein needs to be proteolytically processed by low pH-dependent proteases in the endocytic compartment. To study the effect of NH125 on the activity of viral envelope proteins of highly pathogenic viruses, we made use of recombinant chimeric VSV that expressed the foreign envelope glycoprotein(s) in place of the VSV G gene. In addition, the viruses were engineered to encode the sNLuc reporter protein. Infection of BHK-21 with VSVΔG(H5,N1,sNLuc) expressing both the hemagglutinin (HA) and neuraminidase (NA) of the highly pathogenic avian influenza virus A/chicken/Yamaguchi/7/04 (H5N1) was significantly affected by bafilomycin A1, in accordance with the pH-dependent fusion activity of HA (Figure 7a). As anticipated from a lysosomotropic compound, NH125 inhibited infection by VSVΔG(H5,N1,sNLuc) in a concentration-dependent manner. Likewise, infection of BHK-21 with VSVΔG(EBOV-GP,sNLuc), a chimeric VSV encoding the Ebola virus glycoprotein, and VSVΔG(Lassa-GP,sNLuc), a chimeric virus expressing the Lassa virus glycoprotein, was affected by NH125 and bafilomycin A1.

Several non-enveloped viruses also require an acidic milieu for infection as the low pH is needed to trigger the disassembly or rearrangement of the viral coat proteins. When we analyzed the effect of bafilomycin A1 on infection of BHK-21 cells with bluetongue virus type 8 (BTV-8), a strong reduction of focus-forming units was observed (Figure 7c), in line with previous reports showing that BTV entry is sensitive to bafilomycin A1 [48]. Accordingly, infection by BTV-8 was also sensitive to NH125, showing an even higher sensitivity to this drug than VSV* (Figure 7b).

Most paramyxoviruses have a pH-independent fusion activity and can enter the cell by direct fusion with the plasma membrane. We, therefore, analyzed the effect of NH125 on infection of BHK-21 with two members of this virus family, bovine respiratory syncytial virus (BRSV) and Sendai virus (SeV). Unexpectedly, recombinant BRSV* expressing GFP was highly sensitive to both NH125 and bafilomycin A1 (Figure 7c), suggesting that BRSV infection requires a pH-sensitive step [49]. In contrast, infection of BHK-21 by SeV was not affected by bafilomycin A1, confirming that this paramyxovirus indeed enters the host cell in a pH-independent manner (Figure 7d). Nevertheless, NH125 potently inhibited infection by SeV, suggesting that inhibition of this virus was not mediated by NH125-driven alkalization of the endocytic pathway but relied on a different mechanism. 

## 4. Discussion

The screening of 80 commercially available kinase inhibitors led to the identification of NH125, an imidazolium derivative, which demonstrated antiviral activity against VSV and other viruses. Our data suggest that NH125 is a lysosomotropic compound which may cause alkalization of the endocytic compartment thereby inhibiting VSV G protein-induced membrane fusion and, consequently, virus entry. In particular, at higher concentrations, NH125 showed cytotoxic effects, which are likely a consequence of the lysosomotropic activity of this compound. 

NH125 contains a central imidazolium ring which is substituted at positions 1, 2, and 3 by a benzyl, methyl and hexadecyl group, respectively. In particular, the aliphatic hexadecyl side chain may add to the hydrophobic properties of the compound (see Figure 1d). NH125 has been described as an antimicrobial agent which inhibits bacterial histidine kinases [36,50]. Structural analogies between bacterial histidine kinases and eEF2 kinase led to the assumption that NH125 may inhibit the activity of eEF2 kinase as well. Indeed, NH125 was demonstrated to inhibit the activity of eEF2 kinase in vitro and to inhibit the growth of cancer cells with markedly increased expression of eEF2 [37]. However, these results have been put into question as other studies demonstrated that the inhibition of eEF2 kinase was rather weak and unspecific. On the contrary, NH125 was found to increase eEF2 phosphorylation in cancer cells, thereby reducing protein synthesis levels which, in turn, could account for the anticancer activity of this compound [38,39]. In line with these previous observations, we found that treatment of cells with NH125 caused eEF2 phosphorylation, however, expression and secretion of the sNLuc reporter protein was not affected by NH125. Likewise, in vitro translation of luciferase mRNA was not suppressed by NH125, suggesting that neither protein synthesis nor the secretory pathway were affected by NH125. This observation seems to be in conflict with the observed NH125-mediated phosphorylation of eEF2. However, it is possible that the proportion of phosphorylated eEF2 was too low to have any significant impact on protein synthesis. Consequently, the cytotoxic properties of NH125 must rely on other mechanisms.

NH125 is structurally related to *N*-dodecylimidazole, a known lysosomotropic compound [45], and may act on cells in a similar way. As the acidic milieu of endosomes and lysosomes is important for many physiological processes of the cell, long-term alkalization of these cellular compartments may affect cell viability. It is, therefore, possible that the cytotoxic effects by NH125 are related to this aspect of cell health. However, NH125 may also cause cell death by disrupting the lysosomal compartment as has been suggested for *N*-dodecylimidazole and other lysosomotropic agents [45,51]. The critical NH125 concentration leading to 50% BHK-21 cell death within 24 h was found to be 10 µM (see Figure 6a) while the IC_50_ was about 0.5 µM (see Figure 2a). The selective index (SI) of 20 indicates that the therapeutic NH125 concentration range is rather small. 

In line with its lysosomotropic features, NH125 exhibited broad-spectrum antiviral activity. Not only do many enveloped viruses enter their host cell via a pH-dependent fusion mechanism, but also many non-enveloped viruses have a pH-dependent uncoating process [52], which may be targeted by NH125. Surprisingly, however, SeV was not inhibited by bafilomycin A1 but, nevertheless, was highly sensitive to NH125. This suggests that the inhibition of SeV may not be due to the lysosomotropic activity of NH125 but may rely on a different mechanism.

NH125 shows structural similarity with compounds known as imidazolium-based “ionic liquids”, i.e., salt-like compounds that are liquid below 100 °C [53]. Imidazolium-based ionic liquids are typically composed of an imidazolium moiety, which is substituted with one or two alkyl chains of different lengths. These amphiphilic molecules have surfactant-like properties and were shown to inhibit growth of bacteria, as well as cancer cells [53,54,55]. Imidazolium-based cations were demonstrated to insert into biological membranes, thereby modulating their structural organization and permeability [56,57]. The cytotoxic properties of ionic liquids are closely linked to the alteration of membrane permeability and depend on the length of the alkyl chain with C8 or shorter chains normally showing no cytotoxicity [58,59]. Given the structural similarity between NH125 and imidazolium-based ionic liquids, it cannot be ruled out that NH125 has surfactant-like properties as well. As the composition of the host cell membrane is crucial for entry of enveloped viruses [60], it is, therefore, possible that NH125 is able to cause structural alterations in the plasma membrane or derived endosomal membranes with a consequent negative effect on virus-mediated membrane fusion.

## 5. Conclusions

In summary, we identified a small compound, NH125, which showed antiviral activity against VSV and other viruses. NH125 has previously been described as a specific inhibitor of eEF2 kinase, however, the antiviral activity of NH125 appeared not to be related to eEF2 phosphorylation and did not involve inhibition of protein synthesis. NH125 rather showed lysosomotropic activity, which explains the broad antiviral effects by this compound, but also its cytotoxic properties. In addition, NH125 may have surfactant-like activity, which may affect the integrity of cellular membranes and thereby may interfere with membrane fusion and virus entry. Since NH125 targets cellular processes rather than the virus, the emergence of resistant viruses is unlikely to occur. However, the cytotoxic properties of NH125 may limit the development of this compound into a clinically approved antiviral drug. 

## Supplementary Materials

The following is available online at http://www.mdpi.com/1999-4915/10/6/306/s1, Table S1. List of kinase inhibitors that have been screened for antiviral activity against VSV*∆G(FLuc). Figure S1. Inhibition of VSV entry by lysosomotropic compounds. Figure S2. Effect of chloroquine on BHK-21 cell viability.
