# Supplementary material for: 1-Benzyl-3-cetyl-2-methylimidazolium Iodide (NH125) Is a Broad-Spectrum Inhibitor of Virus Entry with Lysosomotropic Features
Source: Viruses. 2018 Jun 5;10(6):306. doi: 10.3390/v10060306 (PMC6024324; doi:10.3390/v10060306)
Supplement: Supplementary file 1 [file viruses-10-00306-s001.pdf]

Table S1. List of kinase inhibitors that have been screened for antiviral activity against VSV*ΔG(FLuc).

Compound	Target kinase	Inhibition of luciferase (%)[1]	Compound	Target kinase	Inhibition of luciferase (%)[1]
AG 490	EGFR	45.0	10-DEBC	PKB	70.2
ML 9	MLCK	33.1	TPCA-1	IKK	38.2
NH125	CaM Kinase III	97.8	SB 218078	Chk1	13.2
Fasudil	ROCK	20.3	TCS 359	FLT3	6.8
GF 109203X	PKC	80.9	PD 198306	MEK	44.5
Genistein	EGFR	39.8	Ryuvidine	cdk	99.1
LY 294002	PI3K	44.5	IMD 0354	IKK	93.6
U0126	MEK	52.0	CGK 733	ATR/ATM	82.9
PD 98059	MEK	41.5	PHA 665752	cMET	90.5
Y-27632	ROCK	15.2	PD 407824	Chk1	31.9
SB 202190	p38 MAPK	26.3	LY 364947	TGFbR1	4.8
Olomoucine	cdk	29.4	CGP 57380	Mnk1	50.0
LFM-A13	BTK	15.1	PQ 401	IGF-1R	33.1
ZM 336372	Raf	16.9	PI 828	PI3K	12.7
ZM 449829	JAK3	29.1	NU 7026	DNA-PK	29.8
ZM 39923	JAK3	16.5	D 4476	CK1	0
GW 5074	Raf	0.5	EO 1428	p38 MAPK	45.4
PP 1	Src	31.2	H 89	PKA	45.0
SB 203580	p38 MAPK	10.8	FPA 124	PKB	11.3
(-)-Terreic acid	BTK	33.0	GW 843682X	PLK	26.7
PP 2	Src	16.2	Iressa	EGFR	62.4
SU 4312	VEGFR	51.3	SU 5416	VEGFR	21.4
SP 600125	JNK	75.4	1-Naphthyl PP1	Src	4.5
Purvalanol A	cdk	51.8	GSK 650394	SGK	7.1
Purvalanol B	cdk	1.5	BIO	GSK-3	81.7
KU 55933	ATM	53.1	SD 208	TGFbR1	25.5
SB 431542	TGFbR1	23.5	Compound 401	DNA-PK	13.6
SB 216763	GSK-3	36.9	BI 78D3	JNK	13.0
SB 415286	GSK-3	15.1	SC 514	IKK	43.0
Arctigenin	MEK	42.6	Hexabromocyclo-hexane	JAK2	0
NSC 693868	cdk	0	HA 1100	ROCK	0
SB 239063	p38 MAPK	23.8	BIBX 1382	EGFR	0
SL 327	MEK	27.0	CGP 53353	PKC	9.9
Ro 31-8220	Broad	83.6	Arcyriaflavin A	cdk	24.3
Aminopurvalanol A	cdk	51.4	ZM 447439	Aurora	16.8

API-2	PKB	48.3	ER 27319	Syk	81.0
GW 441756	TrkA	18.8	ZM 323881	VEGFR	27.8
GW 583340	EGFR	29.1	ZM 306416	VEGFR	30.4
Ro 08-2750	TrkA	33.7	IKK 16	IKK	98.0
TBB	CK2	0	Ki 8751	VEGFR	6.2

[1] Vero cells were treated for 2 hours with the inhibitors (10 μM) and subsequently infected with VSV*ΔG(FLuc) using an m.o.i. of 0.5 ffu/cell. The cells were maintained in the presence of inhibitor and lysed 6 hours post infection. The percentage luciferase activity relative to DMSO-treated cells is shown (mean value of two 2 independent experiments). Inhibitors suppressing virus-driven luciferase expression by more than 95% are printed in red letters.

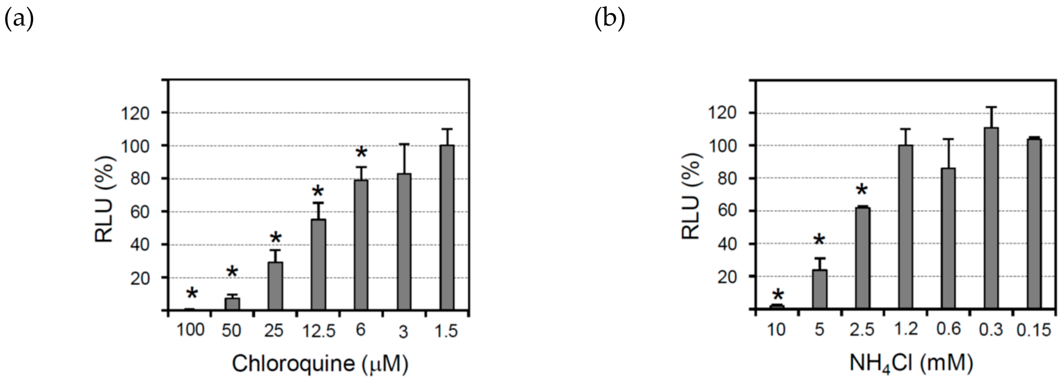

Figure S1. Inhibition of VSV entry by lysosomotropic compounds. BHK-21 cells were treated for 1 hour with either chloroquine (a) or NH_4Cl (b) using the indicated concentrations. The cells were inoculated in the presence of the drugs for 1 hour with VSV*ΔG(sNLuc) using an m.o.i. of 0.5 ffu/cell. The cells were subsequently washed and incubated in the presence of monoclonal antibody I1 to prevent virus entry at later time. Secreted Nano luciferase activity was determined in the cell culture supernatant 6 hours p.i. The percentage RLUs relative to mock-treated cells were calculated. Mean titers and standard deviations of 6 parallel experiments are shown. The asterisks indicate virus entry inhibition at levels significantly different from mock-treated cells.

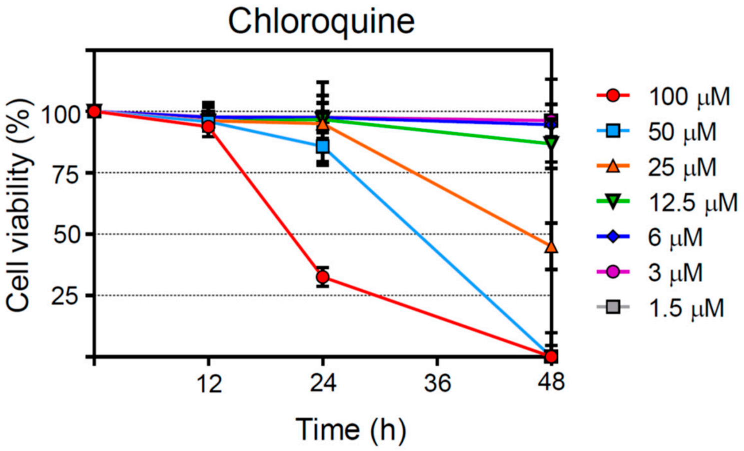

Figure S2. Effect of chloroquine on BHK-21 cell viability**.** BHK-21 cells were incubated with chloroquine using the indicated concentrations. The proportion of dead cells was determined at the indicated times by determining the activity of dead cell protease released from dying cells and in total cell lysates. Mean values and standard deviations of 4 parallel experiments are shown.
